# Urbanisation as driver of food system transformation and opportunities for rural livelihoods

**DOI:** 10.1007/s12571-021-01182-8

**Published:** 2021-06-28

**Authors:** Sophie de Bruin, Just Dengerink, Jasper van Vliet

**Affiliations:** 1 PBL Netherlands Environmental Assessment Agency, The Hague, The Netherlands; 2 Amsterdam, The Netherlands; 3 Institute for Environmental Studies, Vrije Universiteit Amsterdam, Amsterdam, The Netherlands

**Keywords:** Food system transformation, Urbanisation, Rural livelihoods, Enabling conditions, Sustainable development

## Abstract

Urbanisation is changing food systems globally, and in particular in sub-Saharan Africa and South Asia. This transformation can affect rural livelihoods in multiple ways. Evidence on what enabling conditions are needed to materialise the opportunities and limit risks is scattered. Here we review scientific literature to elaborate on how urbanisation affects food systems, and on the enabling conditions that subsequently shape opportunities for rural livelihoods. We find that urbanisation leads to a rising and changing food demand, both direct and indirect land use changes, and often to more complex market linkages. Evidence shows that a wide range of enabling conditions can contribute to the materialisation of opportunities for rural livelihoods in this context. Reviewed evidence suggests that the connectivity to urban centres is pivotal, as it provides access to finance, inputs, information, services, and off-farm employment. As a result, physical and communication infrastructure, the spatial pattern of urbanisation, and social networks connecting farmers to markets are identified as important enabling factors for the improvement of rural livelihood outcomes. Our findings suggest that coordinated and inclusive efforts are needed at different scales to make sure rural livelihoods benefit from urbanisation and food system transformation.

## Introduction

Urbanisation contributes to the transformation of food systems by shaping spatial patterns of food demand and affecting consumer preferences (Tefft et al. 2017; Seto and Ramankutty 2016). This transformation is a multifaceted process, changing market linkages and relations between food system actors (HLPE 2017; Mergenthaler et al. 2009). In addition, urbanisation leads to urban expansion, causing both direct and indirect land-use changes (van Vliet 2019).

The theory of structural transformation describes the co-evolution of urban and rural areas. This theoretical perspective starts from the increase in agricutural productivity in rural areas, leading to some farmers creating a surplus. The additional income from this surplus subsequently generates demand for goods and services thus stimulating the off-farm sectors of the economy (Jayne et al., 2016, 2018). As a result, there is a gradual shift of jobs from the primary agricultural sector to secondary and tertiary sector jobs, typically allocated in urban areas, thus stimulating rural-to-urban migration (Christiaensen and Martin 2018; Davis and Henderson 2003). Hence, the theory of structural transformation essentially frames urbanisation as a result of agricultural development leading to broader economic growth. However, especially in some low-income countries, urbanisation is not neccesarly associated with economic growth but rather with rapid overall population growth and lacking investments in urban infrastructure (Castells-Quintana and Wenban-Smith 2020).

Both in situations of structural transformation and in situations of rapid population growth without structural transformation, urbanisation is shaped by rural developments, and urbanisation in turn affects rural development. A growing urban population affects rural areas via their demand for food, via remittances sent back to rural areas, and through changes in land use and land cover needed to accomodate these people and their activities. These developments can create both opportunities and risks for rural livelihoods (Da Silva and Fan 2017; Agergaard et al. 2019; Skinner 2018). While much has been written about urbanisation and food system transformation in recent years (Battersby and Watson 2018; Hussein and Suttie 2018; Kookana et al. 2020; Masters et al. 2013), there is no shared understanding of the conditions that shape the impacts of urbanisation and associated food system on rural livelihoods.

To address this knowledge gap, this paper first reviews how urbanisation affects food systems, and subsequently explores the enabling conditions that shape how these food system changes affect rural livelihoods. While we acknowledge the reciprocal relationship between urbanisation and rural development, this study focuses on the various impacts of urbanisation on rural livelihoods via changes in food systems. Geographically, we focus on sub-Saharan Africa and South Asia as these regions are projected to be hotpots for urbanisation in the near future while they also face very large challenges in terms of sustainable development, notably related to poverty, food security, and health. Especially rural areas fall behind. In sub-Saharan Africa and South Asia over 80% of the extreme poor and around 75% of the moderate poor live here and the majority of these people depend at least partly on food system activities, mostly on food production (Castañeda et al. 2016).

The findings of the paper are based on a comprehensive qualitative literature review, taking the geographic focus on South Asia and sub-Saharan Africa and the conceptual framework provided in the second section as a starting point. The three central concepts: urbanisation, food systems, and rural livelihoods were the main searching terms to start searching in both Google Scholar and Scopus, followed by snowballing. We focused on the literature that has been published in the past ten years, to include the most recent findings. Yet, we refer to older publications when these are important studies on urbanisation, food systems, and rural livelihoods, and when no more recent study was found on a particular topic. The third section assesses the major changes urbanisation brings about in food systems, including the magnitude of projected future developments. The fourth section discusses five enabling conditions that strenghten the potential opportunities for rural livelihoods: the social, physical, spatial, economic, and institutional conditions. This section also touches upon the interactions between the conditions. The fifth and last section explores the implications for policy and sustainable development.

## Conceptual framework and definitions

**Fig. 1:**
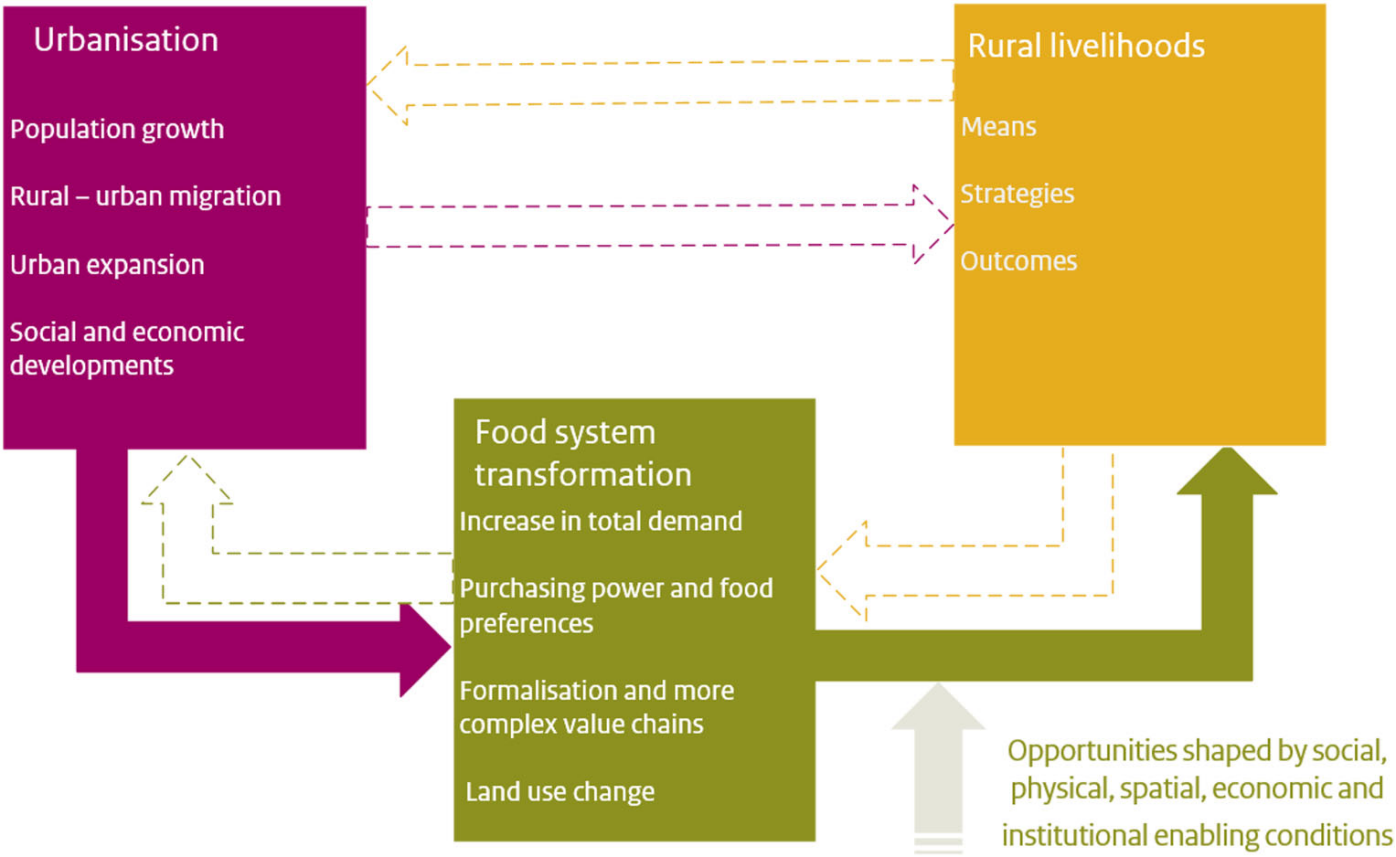
Conceptual framework of this paper. Urbanisation, food system transformation and rural livelihood developments all (re)shape each other. In this review, we focus primarily on the consequences of urbanisation and food system change for rural livelihoods

In this study we zoom in on the processes of urbanisation as a driver of food system transformation, and analyse the impacts of observed and projected changes on rural livelihoods, as conceptualised in Fig. 1. While the strict definition of urbanisation refers to a demographic process leading to an increasing share of the population living in urban areas, we interpret urbanisation more broadly and comprehensively here, including social, economic, and spatial changes as well (see for example Kuang et al. (2020)). In line with this interpretation, urbanisation is not seen as an autonomous process but depending on and (re)shaped by rural developments including population growth and agricultural developments (Agergaard et al. 2021).

The food system concept used in this study underlines how food system activities are embedded in their broader socio-economic and environmental context (Ericksen 2008; HLPE 2017; Ingram 2011; FAO et al. 2020). Although the food system approach has been used for over 30 years, its use and application gained popularity in recent years in the nutrition and food security community (Ericksen et al. 2012; HLPE 2017; Tschirley et al. 2015). The approach supports the identification of trade-offs and synergies between health, food production, farmer income, biodiversity, and climate change, rather than focussing on one of these aspects separately (Ruben et al. 2019; Ericksen 2008). It can be applied at different geographical levels, from local to global.

Food systems that transform, undergo a systemic change. This systemic change often relates to a change from traditional and mostly rural systems based on local market linkages and employment opportunities in farming, to a system with more complex market linkages and more diverse employment opportunities along the food value chain and beyond, such as in research and development (HLPE 2017; Mergenthaler et al. 2009; IFPRI 2015). However, in reality, these transformations are far from linear and very complex in nature.

The livelihood concept has been used in the development practice and research community for over 30 years with different interpretations (Scoones 2009). Here, we define livelihoods as the means and strategies people have to improve their quality of life, i.e. their livelihood outcomes: income, well-being and food security, decreased vulnerability to shocks, and the sustainable use of natural resources (Scoones 2009; Serrat 2017). These livelihood means include for example food, knowledge, and shelter. Livelihood strategies include growing food, selling crops, and finding off-farm employment to achieve livelihood outcomes (Serrat 2017). Rural households often rely on a diverse portfolio of livelihood strategies to achieve better livelihood outcomes (Scoones 2009).

How urbanisation affects rural livelihoods via food system transformations is highly dependent on a series of enabling conditions that contribute to the realisation of potential opportunities. Here, we define enabling conditions as factors that increase the likelihood of improved rural livelihoods outcomes (see for a discussion on definitions of enabling conditions Huber-Stearns et al. (2017)). For this study we identify five categories of enabling conditions, as described in Table 1.

**Table 1:** Categories of enabling conditions used in this study

Enabling condition	Specification	References
Social conditions	Networks, norms, knowledge	Walther et al. (2019), De Brauw et al. (2014), Pingali et al. (2019)
Physical conditions	Infrastructure, food markets, and land availability	Sheahan and Barrett (2017), Skinner (2018), Torero (2014), Weiss et al. (2018)
Spatial conditions	Patterns of urbanisation	Christiaensen and Todo (2014), Dorosh and Thurlow (2013), Tadesse (2012)
Economic conditions	Trade policies and financial incentives	Banerjee and Duflo (2019), Clapp (2017), Pingali (2015)
Institutional conditions	Government services and governance	Abdychev et al. (2018), Candel (2014), Pingali et al. (2019); Vink (2017)

## Impacts of urbanisation on food systems

In this section, we discuss the most important impacts of urbanisation on food systems which, in turn, affect rural livelihoods: the overall growth of urban food demand; purchasing power and related food preferences; increasingly complex food value chains; and direct and indirect land use changes.

### Rising urban populations

Both sub-Saharan Africa and South Asia are projected to face rapidly growing urban populations towards 2050. The ‘middle-of-the-road’ scenario for sub-Saharan Africa projects an urban population of about 840 million in 2050, compared to 261 million in 2010 (UNDESA 2020). In South Asia, the urban population is projected to rise from 545 million to 1.2 billion following the same scenario. Besides the inherent uncertainties with regard to future projections, these numbers have to be interpreted with care, because definitions of urban areas differ between countries and also over time within countries. Moreover, the strict distinction between rural and urban areas is in reality more of a gradient, as many locations combine characteristcs of both rural and urban areas (e.g. van Vliet et al. (2020); Tacoli (2003)).

A growing share of the urban population is projected to live in the large cities (> 1 million inhabitants) in both South Asia and sub-Saharan Africa, as illustrated in Table 2. The projected growth of large cities is a continuation of existing trends (Castells-Quintana and Wenban-Smith 2020). However, a large share of the new urban dwellers is expected to live in peri-urban areas surrounding these large cities (Huijstee et al. 2018). Especially in sub-Saharan Africa, cities are often less compact and less dense than in other world regions (Xu et al., 2019). In other words, the continent is ‘suburbanising’, with inhabitants increasingly living in newly developing neighbourhoods or in roadside towns further away from formal city centres (Tieleman 2020). Hence, it is important to realise that urbanisation also relates to the development of towns and the rise of small and medium-sized cities, most of which are strongly embedded in their rural suroundings (e.g. Chai and Seto (2019); Agergaard et al. (2021)).

**Table 2:** Population development in different city sizes (UNDESA 2018)

Region	City size	Percentage of urban			Total population (million)		
		**2000**	**2020**	**2030**	**2000**	**2020**	**2030**
South Asia	< 300,000	47%	42%	39%	199	298	350
	300,000–1 million	13%	13%	13%	56	90	119
	> 1 million	40%	42%	47%	167	321	421
Sub-Saharan Africa	< 300,000	52%	48%	42%	105	219	289
	300,000–1 million	17%	15%	15%	32	71	101
	> 1 million	31%	37%	43%	65	169	276

From a food system perspective, the notion of city size is of importance since smaller cities often depend more strongly on the agricultural economy than larger cities, and have specific functions in local and national food systems (Hardoy et al. 2019). Yet, national governments in both sub-Saharan African and South Asian countries generally invest less in these smaller cities and tend to favour the capital region and urban deltas, with a variety of advantages including better access to financial assets, import-export licenses and better provision of public service (Henderson 2010; Sahoo 2016).

### Rising demand and changing food preferences

Urban food demand is projected to increase and change due to the growing urban population and the rise in average income. Overall food demand is projected to rise approximately 2.5-fold in sub-Saharan Africa and 1.7-fold in South Asia by 2050 compared to 2010, following a ‘medium’ fertility and economic growth scenario (Tabeau et al. 2019; Van Ittersum et al. 2016; de Bruin et al. 2021). In South Asia, population growth projections are lower than in sub-Saharan Africa, while the projected per capita income growth towards 2050 is higher than in sub-Saharan Africa (de Bruin et al. 2021). Altogether urban food demand is expected to rise two to four times more than rural food demand (Zhou and Staatz 2016; Pingali et al. 2019).

Next to the overall increase in food demand, there are shifts in what type of food is demanded. The diversification of urban diets results partly from the fact that urban food environments are different from rural food environments, with a larger array of food products available, and more diverse places to buy and consume food (Minot 2014; Pingali et al. 2019). This wider range of food options includes unhealthy foods, containing high levels of sugar, salt, fat, and is highly processed, but also diverse and nutritious foods for people who can afford it (Hawkes et al. 2017; Pingali et al. 2019). Urban dwellers from all income groups are more likely to eat outside their homes, and the urban food environments are offering different options (Djurfeldt 2015; Bren d’Amour et al. 2020).

Although food environments shape consumption patters, other social and economic factors are also driving consumer preferences (Tschirley et al. 2015; Popkin 2014; Stage et al. 2010). Evidence suggests that rising incomes of urbanites are the most important factor underlying dietary changes towards more animal products, fruits and vegetables, and oils (Tacoli & Vorley, 2015; Zhou and Staatz 2016; Bren d’Amour et al. 2020). Although purchasing power is on average higher in urban regions, it should be noted that the pattern of poverty decreasing alongside urbanisation is not that evident in many regions of sub-Saharan Africa, compared to most of Asia (Turok and McGranahan 2013; Tacoli et al. 2015). Instead, urbanisation often also comes with increased economic inequality and an increase in the population of urban people in poverty (Christiaensen and Todo 2014; Kanbur and Zhuang 2013; Battersby and Watson 2018). Nonetheless, food security levels are on average higher in cities than in rural areas because of the higher overall purchasing power (Stage et al. 2010; Headey et al. 2018; Tibesigwa and Visser 2016). For example, while 18% of West Africa’s rural population is undernourished, this is 13% among the urban population (van Wesenbeeck 2018). Evidence from Ethiopia shows that dietary diversity is also higher in urban areas than in rural areas, and affordable for more people (Gebru et al. 2018). Yet, this does not hold for all countries. For example, in South Africa the share of food insecure households is higher in urban areas than in rural areas (Stats SA 2019). Overall, most rural and urban people in sub-Saharan Africa and South Asia cannot afford the healthy and diverse diet as proposed by the EAT-Lancet commission (Hirvonen et al., 2019; Sharma et al. 2020).

Altogether, the demand for various groups of food products is expected to increase in both sub-Saharan Afica and South Asia, as shown in Table 3. The larger increase in fruits and vegetables, as well as meat products is related to the expected increase in wealth, which for a large part relates to urbanisation. Besides a shift in product types, the consumption of processed foods is expected to rise substantially, especially in the large metropolatian areas (Bren d’Amour et al. 2020; Zhou and Staatz 2016).

**Table 3:** Projected food demands for three food groups in South Asia and sub-Saharan Africa (IFPRI 2017)

Food product group	Region	Total demand (million tonnes)		Index (2010 = 1)	
		**2010**	**2050**	**2010**	**2050**
Fruits & vegetables	South Asia (ex. Iran)	188	901	1,0	5,0
	Sub-Saharan African	102	321	1,0	3,1
Meat	South Asia (ex. Iran)	10	42	1,0	4,3
	Sub-Saharan African	11	48	1,0	4,2
Cereals	South Asia (ex. Iran)	283	479	1,0	1,7
	Sub-Saharan African	141	337	1,0	2,4

### Changing market linkages

An increasing overall demand for food as well as a rising demand for more diverse and processed food creates larger markets, providing new opportunities for millions of farmers, processors, and traders (Jayne et al. 2018). Historically, farmers that are well-connected to urban markets or storage/processing facilities, are more productive and relatively well served by agribusinesses (Masters et al. 2013; Swain and Teufel 2017; Sharma 2016). This can be linked to the observation that farmers close to urban markets are mostly receiving higher returns on their agricultural products due to better access to and information about the growing markets, and lower transaction costs (Diao et al. 2019; Tadesse 2012). Yet, returns are lower for farmers in the rural hinterlands of smaller cities and towns as compared to farmers in the vicinity of large cities (Vandercasteelen et al. 2018). At the same time, millions of smallholder farmers in less accessible or detached hinterlands remain cut off from the opportunities growing urban food markets can bring (Djurfeldt 2015). This difference in opportunties is not only tied to geographic characteristics, but also to gender barriers, social exclusion, trade policies and political decisions.

Urbanisation has in some cases led to more formal and more complex market linkages, as a result of the changing infrastructure needed to connect consumers with producers. More complex markets linkages involve more actors, such as brokers and processors, between farmers and consumers (Debonne et al. 2021a). Some rural and peri-urban households living close to cities benefit from these market linkages, as it provides opportunities to diversify their incomes in the lengthier value chains, such as in processing and transporting of agricultural products (Diao et al. 2019; Djurfeldt 2015; Afriyie et al. 2014). Employment in the off-farm agrifood system is currently growing more rapidly in sub-Saharan Africa than employment in farming itself (Allen et al. 2018). However, this growth started from a lower base, which means that the absolute contribution to new jobs is still higher on-farm than off-farm. Yet, while food system transformation can provide more jobs, especially in post-harvesting and agricultural services, this will not deliver all of the 20 million new jobs that are projected to be needed annually towards 2040 in sub-Saharan Africa (Abdychev et al. 2018).

The vast majority of food traded in sub-Saharan Africa is through informal channels – outdoor markets, kiosks, and street vendors – yet the share of formalised markets is rising in many regions (Porter 2017; Battersby 2017; Reardon et al. 2015). In South Asia, formal markets developed slightly earlier than in sub-Saharan Africa, although informal outdoor markets, kiosks, and street vendors also dominate here (Dakora 2012; Reardon and Minten 2011; Skinner 2018). Battersby (2017) shows that in South Africa, low-income groups have not profitted from more formal food value chains, as these often negatively impact small producers and local businesses. Although some countries in sub-Saharan Africa, notably Kenya and South Africa, as well as large parts of South Asia faced a growth in formal markets, in most places, informal markets are expected to remain in place (Skinner 2018; Pingali et al. 2019; Neven et al. 2009). But in places where formal value chains expand, as in South Africa and Kenya, this process affects prices, quality and safety standards, often restricting access to sale channels for small producers (Jayne et al. 2010; Nickanor et al. 2021). A shift from informal market linkages to value chains implies stricter contracts and delivery schedules (Crush and Caesar 2014; Pingali et al. 2019; Barrientos and Visser 2013). When food markets formalise, this will bring changes in the production and retail process, touching upon questions of equity and inclusion. The roles of small farmers change in a more formal and complex food system, especially when requirements with regard to efficiency and minimal purchase value of volume per order change. Some have argued that when striving towards a more formal food system, small farmers have little future (Collier and Dercon 2014), while others stress that empowering small farmers iskey for sustainable development (IFAD 2015; Wiggins et al. 2010).

### Changing land-use dynamics

Urbanisation influences land-use, which again affects rural livelihoods. Especially in rural regions close to cities, the conversion of agricultural land to urban land affects the livelihoods of people living in or depending on these lands (Smit 2016; Dapilah et al. 2019; Marshall and Randhawa 2017). As agricultural land around cities converts to urban land, this is compensated by the development of new cropland in more remote areas, mostly leading to a loss in natural areas (van Vliet, 2019). Moreover, urban expansion poses risks to land tenure security for farmers, especially in the urban fringes, because agriculture is often not considered a priority by spatial planning policies in sub-Saharan Africa and South Asia and because land rentals get too high (Mpofu et al. 2018). In addition, legal plurality often leads to different interpretations of customary land rights by planning authorities and farmers (Magigi and Drescher 2010).

Urban land expansion affects overall food production, and will continue to do so. Van Vliet (2019) shows that the area of urban expansion was relatively low in sub-Saharan Africa (1.9 Mha) and South Asia^1^ (2.4 Mha) between 1992 and 2015, as compared to the global total of about 38 Mha. In South Asia, over 75% of this urban expansion took place on cropland, whereas urban expansion into cropland was less than 40% in Sub-Saharan Africa. The corresponding equivalent loss in cereal production was approximately 1.1 Mton in sub-Saharan Africa and 7.1 Mton in South Asia per year. Between 2000 and 2030 all of Asia is expected to lose about 3% of its cropland to urban expansion, resulting in a 6% production loss (Bren d’Amour et al. 2017). In Africa, the effects are tripled: a 3% cropland loss translates into a 9% crop production reduction in the same period, most of which will take place in Egypt and Nigeria (Bren d’Amour et al. 2017). This leverage effect is because agricultural land around cities is often more fertile, an important reason why cities historically developed in these locations, and also because land management intensity in these areas is typically higher, leading to smaller yield gaps (Vandercasteelen et al. 2018; Gibson et al. 2017).

Urbanisation has different impacts on farm sizes and therefore on rural livelihoods, depending on land tenure security, non-farm opportunities, and the magnitude and impact of land purchases by urban buyers (Masters et al. 2013; Swain and Teufel 2017). Rising population numbers have led to a decrease in farm sizes in low-income countries from on average 2.5 ha in 1960 to on average 1.5 ha in 2000. With less land available per family, familiy members often search for off-farm employment opportunities, often in cities (Lowder et al., 2016). Asia has now passed the turning point where average farm sizes cease to decline, while in Africa average farm sizes are expected to continue to fall, posing challenges in both hinterlands and commercialised areas (Masters et al. 2013). In parallel with this development, urbanites increasingly acquire farm land in sub-Saharan Africa, which contributes to the increase of average farm sizes (Jayne et al., 2016), although these dynamics differ between regions (Debonne et al. 2021b). The growth of medium-scale farms in for example Zambia and Nigeria is partly attributable to land acquisition by salaried urbanites and exacerbates rural income inequality (Sitko and Jayne 2014; Muyanga et al. 2019).

Another dynamic that is related to distant and often urban consumers is the increase in large-scale land acquisitions (LSLA) that have emerged in India and Zambia as well as elsewhere (Narain 2009; Chu et al. 2015). Rural impacts of LSLA are mixed: negative consequences in terms of land appropriation are well documented and affect rural livelihoods in terms of displacement or loss of income. On the other hand, LSLA may provide opportunities in terms of employment, although mostly low-paid. The overall implications for rural livelihoods will differ per situation, although Jayne et al. (2016) conclude that it will likely reduce the rural impacts of agricultural growth and local spill-overs to the rural non-farm economy, and thus reducing opportunities for rural livelihoods.

Besides actual farm size, urbanisation can affect land-use practices in terms of intensification and diversification (Swain and Teufel 2017; Dorosh et al. 2012; Tadesse 2012). These trends are driven by improved access to (urban) food markets and access to inputs and services. However, patterns of agricultural intensification and diversification around urban centres are not homogenous. Steinhübel and von Cramon-Taubadel (2020) show that proximity to smaller cities stimulates the uptake of modern agricultural inputs in India. For Bangalore, this effect is hardly found. The authors suggest that off-farm employment may yield more opportunities to farmers close to this metropole than increasing agricultural inputs. Another counter example is given by Diao et al. (2019), who show that agricultural areas close to larger cities do not necessarily use more inputs than areas further away from urban areas in Ghana.

## Enabling conditions shaping impacts on rural livelihoods

The potential opportunities for rural livelihoods are shaped by enabling factors: social, physical, spatial, economic, and institutional conditions that increase the opportunities for rural livelihoods. Here we review the enabling conditions that have been identified in recent literature.

### Social enabling conditions: Networks, norms, and knowledge

Social enabling conditions include the bonds that connect people in rural and urban areas, social protection measures. and the norms and knowledge that shape the behaviour of urban consumers and the knowledge and skills that allow rural actors to respond to this urban demand.

Social networks are shaped by, among others, migration flows, connecting rural and urban regions through social and business relations, in addition to the financial benefits of remittances (Crush and Caesar 2017; Scheffran et al. 2012). Scheffran et al. (2012) show that migration dynamics contribute to social capital by increasing social resilience in the communities of origin and contribute to the transfer of knowledge, remittances and other resources. However, concerns of emigration for rural areas include a loss of vital workforce and a skewed composition of the population, since mostly young people migrate (Bisht et al. 2020).

Social networks between urban and rural areas allow producers to adequately adjust their production to a changing urban demand and to grasp urban employment opportunities (de Bruin and Dengerink 2020). Walther et al. (2019), for example, show that well-connected food system actors in Niger, Nigeria, and Benin have higher earnings. Consistently, commercial success depends partly on social capital, the norms and networks that people have which enables them to act collectively (Woolcock and Narayan 2000). Social capital is of special importance in cross-border trade, which is characterised by uncertainty in terms of prices, reliability of trading partners, and state positions on imports and exports (Walther et al. 2019). Social protection measures can stimulate agricultural productivity and can reduce (rural) poverty and food insecurity by improving incomes and coping with risks (Croppenstedt et al. 2018; Tirivayi et al. 2016). Especially small farmers are vulnerable to environmental and economic risks, which often leads to risk-avoiding livelihood strategies. These strategies can subsequently reduce their income potential, because of low use of inputs, resulting in a continuation of the status quo or even further deprivation. Investment in social protection are lowest in South Asia and sub-Saharan Africa (Lowder et al. 2017). In these rural areas, the share of the poorest quintile receiving some sort of social assistance is about 27% and 22% respectively, compared to 72% in Latin America and the Caribbean, for example (Lowder et al. 2017). Jones et al. (2017) and Hidrobo at al. (2017) find that by improving structural social protection measures, such as insurances and cash transfers, farmers can be enabled to raise productivity and incomes, thus improving rural livelihoods and reducing food insecurity and poverty.

Evidence suggests that increasing knowledge of urban dwellers on the economic and health advantages of buying local vegetables, fruits, and cereals, can benefit surrounding rural areas due to increasing demand for these products. For instance, studies performed in Kenya and Tanzania show that the promotion of indigenous vegetables can boost rural economies and improve health and environmental outcomes, while also benefiting rural livelihoods (Rampa and Knaepen 2019; Bizzotto-Molina et al. 2020). Yet, increasingly, urban norms about preferred foods are determined by media and its advertising, often steering urban consumers towards unhealthy food options (Pingali et al. 2019).

Garforth (2011) analyses the different types of knowledge and information farmers need to satisfy the larger and changing demand for food. These are first, information on current and new technologies; second, access to business advice; third, information on markets, including timely information on prices; and last, information on domestic policies and regulations. This information can be obtained by informal communication via social networks, non-state organisations such as farmers associations, commercial enterprises, and via the state (Garforth 2011). An interesting example of a social network is the East-African digital platform Mkulima Young, where rural farmers can offer their agricultural produce and keep up to date with the urban demand for agricultural products. Irungu et al. (2015) show this platform has not only connected rural supply with urban demand, but also engaged more youth to become active in agriculture.

### Physical enabling conditions: Infrastructure, food markets, and land availability

Whether rural livelihoods can benefit from urbanisation is largely dependent on physical conditions: the state of communication and transport infrastructure that connects rural and urban areas, the infrastructure around food markets, the availability of fertile land, and water infrastructure.

Several studies show that investments in rural transport and communication infrastructure play a key role in bringing down the transaction cost for farmers and traders, and in improving the quality and freshness of local produce, while stimulating productivity (Hussein and Suttie 2018; Berg et al. 2016; Torero 2014). Consistently, Dorosh et al. (2012) demonstrate that in sub-Saharan Africa, the adoption of high-input technology and crop productivity is higher when producers live closer to urban centres, further demonstrating the importance of accessibility. Stifel and Minten (2008) observe similar dynamics for Madagascar, where they find a strong negative relation between levels of isolation, in terms of travel time and transport costs to the nearest primary urban center, and agricultural productivity as well as welfare. Thereby, higher prices for inputs in isolated areas often make households invest little in their land, but rather expand into less fertile land (Chamberlin et al. 2014; Stifel and Minten 2008). Finally, Stifel and Minten (2008) find that more isolated households underinvest resources in their agricultural land when the benefits are uncertain in the presence of violence and other forms of insecurity.

**Fig. 2:**
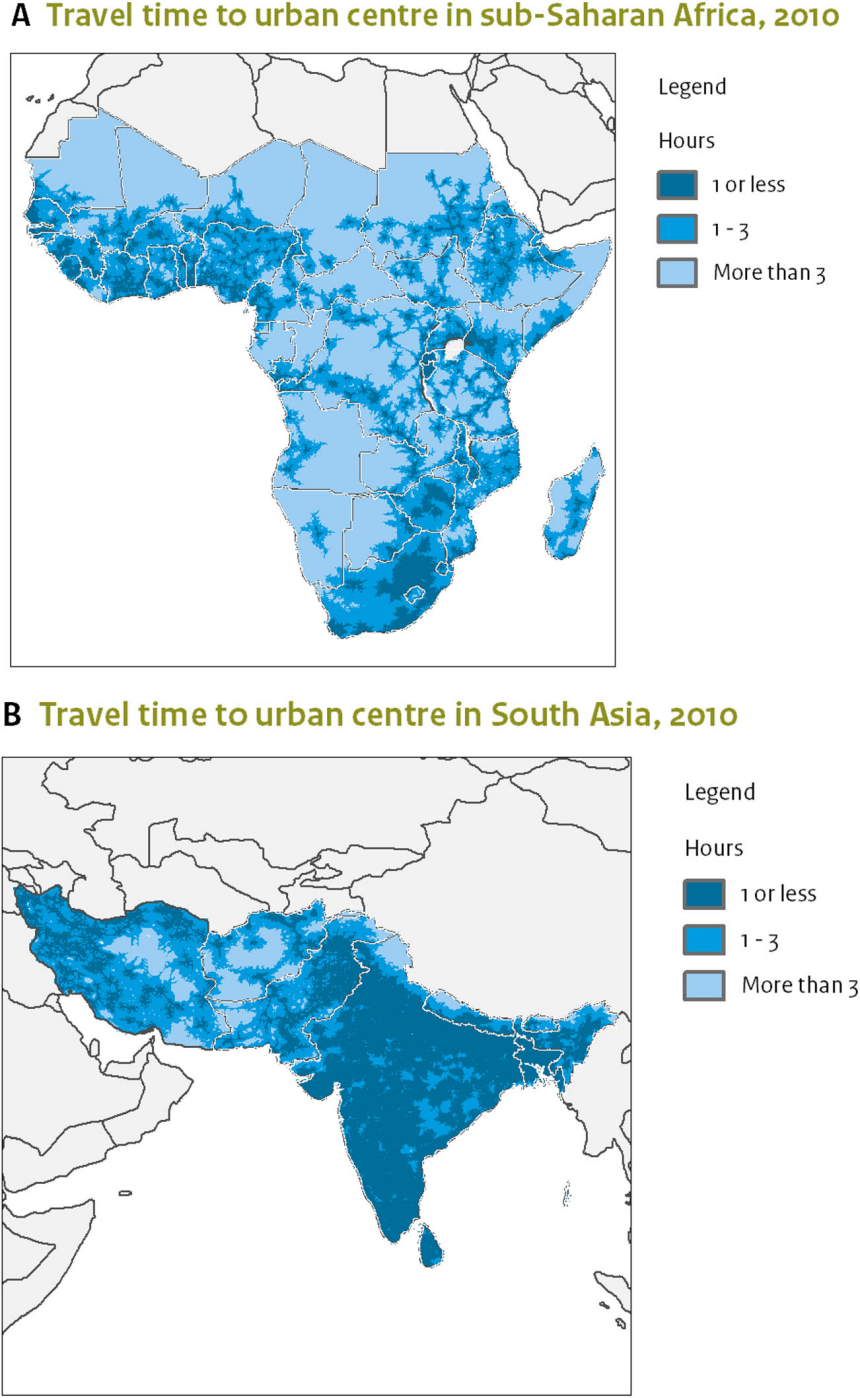
Travel times to urban centre in 2010 in A. sub-Saharan Africa and B. South Asia (Huijstee et al. 2018)

Travel times differ widely between countries in sub-Saharah Africa and South Asia. In India and Nigeria, the majority of the population lives within an hour of a city (Weiss et al. 2018). Following Weiss et al. (2018), on average half of the people in low-income countries lives further than an hour away from a city but most people have to travel less than three 3 h to reach a city. Figure 2 illustrates which regions were well-connected to urban centres in 2010, as well as the regions that are more than 3-h away from a city center. Especially in sub-Saharan Africa, there are large regions which are not well connected to urban areas, although these regions are mostly sparsely populated (Huijstee et al. 2018).

Communication infrastructure can strengthen rural-urban linkages and provide up-to-date market information. For example Bahrini and Qaffas (2019) show that mobile phone and internet adoption are key factors driving economic growth in sub-Saharan Africa (Bahrini and Qaffas 2019). Torero (2014) also shows for example that farmers’ access to (digital) information on markets prices via internet connections tend to have a positive impact on farmers income. However, the better existing information streams, the more specific information is needed for farmers to raise their incomes, especially for farmers producing high-value crops.

The livelihood opportunities for farmers that come with diversifying and rising urban food demands, require investments in logistics, packaging, storage, cooling and processing facilities as well as the physical marketplaces in towns and cities (Reardon et al. 2015). Both pre-harvest facilities, including financial services and the possibility to buy inputs and equipment, and post-harvest facilities, including collection hubs, (cooled) storage, distribution or processing centres, are important (Allen et al. 2018; Dorosh and Thurlow 2013). Access to storage brings an additional advantage for farmers as they can increase their revenues by benefitting from seasonal fluctuations in prices, if they are able to wait (Sheahan and Barrett 2017). Relatedly, Torero (2014) underlines the importance of energy generation in sub-Saharan Africa, since up to 20% of general sales get lost in the informal sector due to energy shortages, in all sectors. The problem of energy shortages is also present in South Asia although less severe: in 1970 South Asia had almost three times the energy generating capacity per person of sub-Saharan Africa, while in 2000, capacity was almost twice as high (Torero 2014).

Physical food markets play a key role in connecting rural production with urban demand. Across sub-Saharan Africa, cities are planning and building marketplaces or upgrading existing ones with proper sanitation, storage and lighting (Minten et al., 2017). Yet, while these formalised markets have advantages in terms of food safety, fees for stalls in upgraded markets are often expensive, decreasing accessibility for most producers and traders. Since nearly all smallholder farmers, most traders in agri-commodity markets and many micro- and small-scale food processors and food retailers are not part of the formal food economy in sub-Saharan Africa (Robinson and Yoshida 2016), improvements in formal markets will not profit these actors. Skinner (2018) shows that policies – and practices – of national and local African governments tend to exclude, evict and relocate informal food markets or sellers, affecting urban food security and access to urban markets for rural actors. Investing in facilities and spatial planning for informal markets is therefore at least as important as investing the formal markets. Understanding how these informal market linkages could be best sustained is also of critical importance, although this knowledge is often lacking (Crush and Young 2019; Resnick 2017).

Poor water quality results from industrial activities and untreated urban waste water and is a central issue for farmers dependend on flood irrigation. Today, less than 10% of all wastewater is treated in developing countries, and if treated, sludge is often dumped (Kookana et al. 2020; Bricas 2019). Water pollution and nutrient accumulation results in diverse risks including human and animal health issues, detoriation of soils as well as plant diseases and contamination (Kookana et al. 2020). The lagging development of wastewater treatment in sub-Saharan Africa and South Asia is projected to dramatically increase nutrient discharge towards 2050, even in the more positive scenarios (Van Puijenbroek et al. 2019). If collected properly in sewerage systems, Nitrogen and Phosphorus collection in both rural and urban areas may yield up to 10% of agricultural demand. The reclosure of nutrient cycles is therefore a key urban food policy challenge, linking urban growth with rural agriculture. A successful example of this is Safi Sana, a social enterprise in Accra, Ghana, which utilises innovative waste-to-resource factories to convert organic and faecal waste into electricity, organic fertiliser and irrigation water (Rao et al. 2017).

Demand for water increases from both the rural and the urban side, especially in parts of South Asia (Ahluwalia 2016; Kookana et al. 2020). Since urbanisation will lead to a higher food demand, rural water use is likely to rise, as it is required for irrigation (Kookana et al. 2020; Ligtvoet et al. 2018). Today, irrigation is limited in sub-Saharan Africa, whereas in South Asia dependence on irrigation is larger, resulting in (ground)water overuse in many regions (de Bruin et al. 2021). Besides water overuse, projected climate change impacts will increase water stress, especially in large parts of South Asia (Ligtvoet et al. 2018). Additional investments in sustainable irrigation and climate adaptation in both regions can decrease vulnerability to changing weather patterns and can can support year-round harvesting of crops.

The physical availability of arable land itself is also an enabling condition shaping the opportunities for rural livelihoods. In Nigeria and some other densely populated countries, such as Uganda, Burundi and Rwanda, little additional arable land is available (Tabeau et al. 2019). This affects the land rush currently taking place in these countries, which is further accelerated by relatively wealthy urban families who acquire land in rural areas, attracted by the expectation of high returns on land and favourable policies (Nolte and Sipangule 2017). Although land is still amply available in most other countries in sub-Saharan Africa, this land is generally less fertile than the land already in use for agricultural production (Doelman et al. 2018), while urban epxansion itself is disproportionally affecting fertile and productive land (van Vliet, 2019). In South Asia, limited availability of arable land is especially a problem in Afghanistan, Bangladesh and India, here, agricultural land already occupies over 50% of the land available (Srinivasa Rao et al. 2016). However, land scarcity is high in most of South Asia due to the relatively high population pressure on the land (Srinivasa Rao et al. 2016). Hence, limited availability of land can disable opportunities for rural livelihood improvements, especially when intensification options are limited.

### Spatial enabling conditions: Patterns of urbanisation

**Fig. 3:**
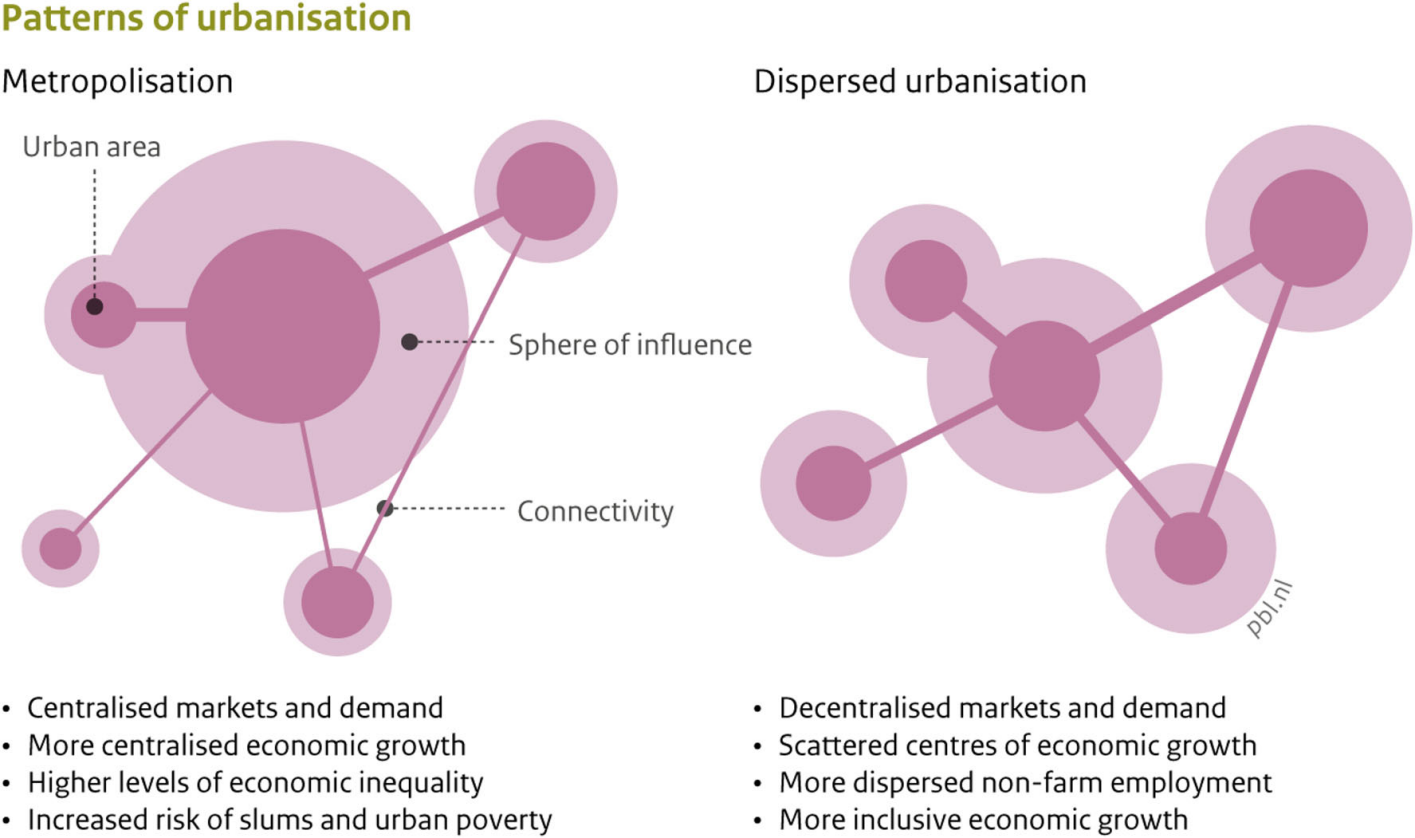
Conceptualisation of urbanisation patterns (taken with permission from de Bruin & Dengerink, 2020)

The spatial patterns of urbanisation, in combination with the quality of infrastructure, shape rural-urban dynamics and rural access to urban markets. A dispersed pattern of urbanisation implies that more smallholder farmers have physical access to food markets, input and knowledge as well as to off-farm employment (Henderson 2010; Christiaensen et al. 2013). The growth of smaller cities rather than the primary cities is correlated with higher levels of poverty reduction by displaying more inclusive growth patterns (Gibson et al. 2017; Imai et al. 2018; Christiaensen and Todo 2014). Two divergent spatial patterns of urbanisation and the major impacts are conceptualised in Fig. 3. Especially the large cities in sub-Saharan Africa and South Asia are projected to grow (UNDESA 2018), which could significantly weaken the future urban growth – falling poverty linkages.

Small and medium-sized cities have important functions for both the rural hinterlands as well as the larger cities (Karg et al. 2019; Steinhübel and von Cramon-Taubadel 2020). Local availability and accessibility to market infrastructure and facilities/services stimulates local labour markets (Allen et al. 2018), affecting the attractiveness of small- and medium-sized cities (Agergaard et al. 2021). In the Uganda Vision 2040, secondary cities (e.g. regional capitals) are envisioned to improve their infrastructure including utilities and transport infrastructure to connect with their hinterlands and enhance regional trade links (FCA 2016). Another example is given by Tadesse (2012), who provides an extensive case for the importance of small cities and towns to rural livelihoods in Ethiopia. The study shows that nearby towns positively influence the ability of households to access markets for selling their crops at a lower cost and for buying inputs, especially fertilizers. This contributes to raising productivity and efficiency, which in turn increases incomes. Thereby are transportation and communication services in towns and small cities helping rural dwellers to access non-farm jobs.

### Economic enabling conditions: Trade policies and financial incentives

Economic conditions, in terms of trade policies and financial incentives, shape the opportunities for rural areas resulting from urbanisation and food system change. Trade policies and financial incentives affect the balance between potentially rising food imports and a consolidation of the domestic agricultural sector. Yet, what the most favourable conditions are in economic terms remains under discussion, as the answer is shaped by ideological beliefs, mixed evidence, and differing interests (Banerjee and Duflo 2019).

Trade policies can stimulate the development of domestic market linkages, as it avoids harming food security as a result of import and export restrictions (Clapp 2017). Low-income countries that rely heavily on food imports are vulnerable to the volatility of world food prices: The soaring international market prices and resulting urban riots that took place between 2008 and 2010 showcased this vulnerability (Bricas 2019). Another example is the influence of food ‘dumps’ from subsidised producers in European countries. These lowered world food prices in the 1990s and 2000s, and reduced incentives to raise local productivity in many low-income countries (Vorley and Lançon 2016; Bureau and Swinnen 2018).

To stimulate the domestic agricultural sector countries can impose temporary import restrictions or increase import tariffs to encourage a supply response and to diminish the extremes of international price volatility (Chang 2009). Some large-scale experiments, such as Nigeria’s Agriculture Transformation Agenda (ATA), use border measures alongside other instruments to give a stimulus to domestic production and rural livelihoods (Vorley and Lançon 2016). Nigeria launched the ATA in 2012 to reduce food imports by increasing production of five key commodities, including rice, sorghum, and cassava, and rationalising the tiers of government to better support private sector agricultural growth. However, there are complex trade-offs involved. Temporarily closing borders for food trade can increase food prices harming food security levels of consumers, although it can make countries also more resilient to high world food prices in the long run. Clapp (2017) shows that there is not one best practice when it comes to improving food security and livelihoods in rural communities, but that there is an array of options between free trade and fully restricting imports, depending on the national context.

A second economic enabling condition refers to agricultural subsidies. Today, subsidies on cultivating staples contribute to unbalanced diets by keeping market prices low compared to other products, resulting in relative high levels of cereal consumption (Micha et al. 2020; Pingali 2015). Jones et al. (2014) show that in Malawi, farm production diversity contributes to household dietary diversity, besides higher and more stable incomes. The authors use these findings to criticise the exclusive financial support for maize cultivation in Malawi: even though this has been effective at increasing national maize production, undernutrition and food insecurity have remained high. More balanced subsidy systems can enable producers to respond to changing urban food demands, and additionally contribute to more positive nutritional outcomes in non-farming households (Pingali 2015).

### Institutional enabling conditions: Government services and governance

Institutional conditions form an integral part of food systems: ‘good’ governance, including the presence of stable institutions play a key role in making sure that rural communities can benefit from urbanisation (Candel 2014; Trebbin 2014). Conflict, lack of institutional capacity, land tenure insecurity, poor policy design, and slow implementation can seriously harm the production, distribution and consumption of healthy food (Candel 2014; Lele et al. 2013). Issues with regard to unequal power distribution among stakeholders decreases their access to information and resources and the ability to exercise influence — especially for smallholder farmers, decreasing equitable and sustainable outcomes (Lele et al. 2013). Institutional characteristics differ widely between countries and tailor-made responses as a lasting part of projects and investments that contribute to food system transformation are essential (Vink 2017).

Access to government services can play a role in reducing risks for rural economies (Pingali et al. 2019; Kosec and Resnick 2019). Effective urban planning, making coordinated infrastructure investments and improving urban transport, could help mitigate risks for rural livelihoods associated with urbanisation (Abdychev et al. 2018). Government policies that improve farmer access to credit, inputs and knowledge can play a key role in improving farmers’ productive capacity to respond to the increasing and changing urban demand. For example, in Meru, Tanzania, urbanisation has stimulated the demand for milk, a reliable source of income for smallholders in a region facing (fertile) land scarcity (Hillbom 2011). Access to inputs, backed up by stable institutions were important conditions for intensification, resulting in higher incomes. Also in the rural regions close to the expanding city region of Delhi, some farmers profit from rising fruit and vegetable consumption in the Indian capital. Although some farmers are profiting, welfare increases are not equally distributed since land ownership and access to markets are rather unequal (de Bruin et al., 2021).

A key element of improved governance is enhanced land tenure security to enable land owners and renters to make durable and sustainable investments in land and to protect peri-urban farmers from losing their land (Holden et al. 2011; Sulieman 2015; Benjamin 2020). Land tenure insecurity sometimes restricts people to (temporarily) migrate for off-farm employment, because people that hold use rights to their land do not always have the right to rent out this land, so they can lose their land if they leave the village (De Brauw et al. 2014). In regions where land rental markets are in place, they underperform in terms of return on long-term investment, as Muraoka et al. (2018) shows for Kenya. Deininger et al. (2019) find that in Malawi tenure insecurity is high amongst farmers and constrains investment in land quality. Agricultural productivity is 9% lower for female operators in Malawi who face tenure insecurity. Similar findings have been observed in Ethiopia (Holden et al. 2011). Having insights into the magnitude of losses in productivity allows for cost-benefit analysis of public programs improving tenure security: Deininger et al. (2019) argue that for the Malawian case benefits can exceed costs sufficiently enough to license such public investments.

## Discussion and implications

The ongoing dynamics of urbanisation in sub-Saharan Africa and South Asia transform food systems in multiple ways. A growing urban population results in growing urban food markets, and a rising demand for diverse products, including more high-value and processed products. Food markets can become more complex due to rising and more diverse demand, and this demand is still largely met by informal market linkages in sub-Saharan Africa. In South Asia, informal markets also dominate, although formalised value chains are more common. The changes in food systems following differing processes of urbanisation can affect rural livelihoods both positively and negatively. The capacity of rural households to act upon opportunities depends on a wide range of enabling conditions which are summarised in Table 4.

**Table 4:** Social, physical, spatial, economic and institutional enabling conditions and the general implications for policies

	**Enabling condition**	**Implication for policies**
**Social enabling conditions**	Strong rural-urban social networks	Stimulating social capital development; facilitating migration
	Social norms and preferences for local food	Public and private marketing of locally produced foods
	Improved knowledge of rural food system actors about urban preferences	Capacity building; access to information channels; facilitating rural-urban social networks
**Physical enabling conditions**	Access to rural-urban transportation and communication networks	Investments in communication and transportation infrastructure and affordable public transport
	Formal and informal market infrastructure: logistics, packaging, storage, cooling and processing	Investment in market linkages: expanding value-adding activities
	More strategically located and better equipped marketplaces	Public investment in formal and informal markets and strategic urban planning of marketplaces
	Availability of arable land	Fair and inclusive land tenure policies, off-farm employment opportunities
	Water treatment and irrigation systems	Investments in water treatment facilities and possibilities for water re-use; further development sustainable irrigation infrastructure
**Spatial enabling conditions**	Dispersed urbanisation patterns	Urban planning stimulating dispersed patterns of urbanisation
	Growth of small- and medium-sized cities	Programs expanding/improving government services in smaller cities, inclusive urban planning
**Economic enabling conditions**	Trade policies that stimulate local production and inclusive trade	Additional taxation of imports which are subsidised in country of origin; subsidies for domestic products
	Agricultural subsidies to stimulate diverse production	More balanced subsidy systems for diverse food production, rather than a sole focus on staples
**Institutional enabling conditions**	Access to government services in smaller cities and rural areas	Investing in access to government services and government capacity in rural and smaller urban areas
	Inclusive land governance	Investing in fair land tenure security, enabling access to institutions

**Fig. 4:**
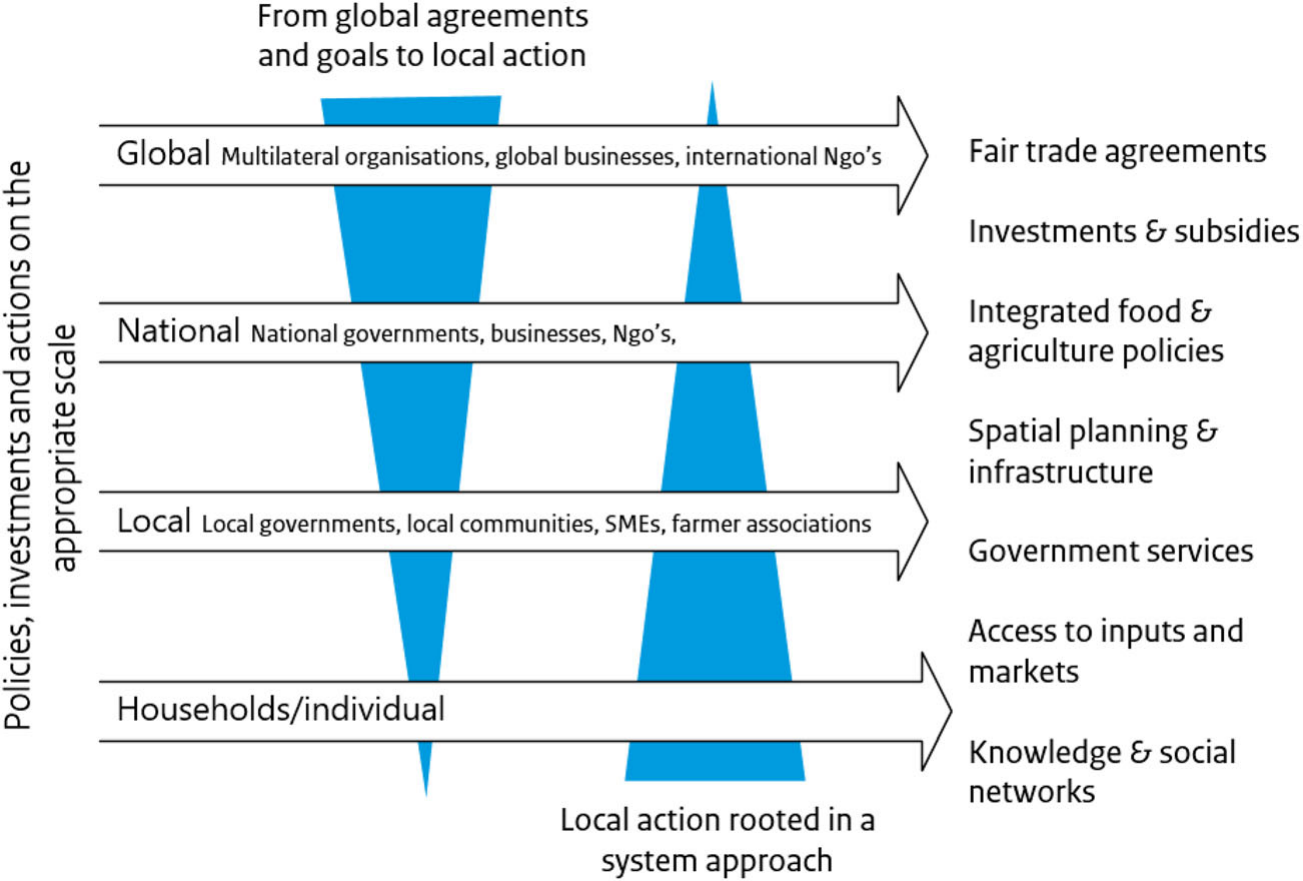
Graphic representation of required actions on different scales to enable rural livelihoods to profit from urbanisation and food system transformation

The array of enabling conditions (summarised in Table 4) that can contribute to sustainable rural livelihoods link to each other on multiple scales and mostly work in conjunction with each other. To make urbanisation work for rural livelihoods, an integrated approach is needed on different levels, which addresses multiple scales and various actors. Figure 4 provides a preliminary overview of scales and appropriate policies, actions and investments. Per situation, there are different bottlenecks that hinder rural livelihoods to benefit from urbanisation. Both global and local action is required, from investments in capacity building programmes and facilitating access to information and inputs on the individual level to national spatial development plans and inclusive trade agreements on the global level. Governments have the power to create or facilitate the enabling conditions, but also to cause damage, as do private and bilateral donors and investors.

The issues of inequality and inequity overarch the influence of enabling conditions. High levels of economic and gender inequality hamper economic growth, food and health security, and potentially contribute to environmental degradation (Doyle and Stiglitz 2014; Kim 2008; Kolawole et al. 2015; Lakner et al., 2020). The poorest farming households are generally less able to benefit from innovations, interventions and value chains (Franke et al. 2014; Ritzema et al. 2017; Rao and Min 2018), making development efforts and investments less effective under high levels of inequality. Therefore, reducing inequalities and inequities in terms of education, public services, income and employment requires continuous consideration in policies, development projects and investments. However, inequality has been rising in recent years and may continue to rise in large parts of sub-Saharan Africa and to a lesser extent in South Asia (Alvaredo et al., 2018; Rao and Min 2018). Today, the economic impacts of Covid-19 are not fully clear, but existing inequalities within countries are increasing, especially between high-income groups and the lower income groups (Egger et al. 2021). Without specific emphasis on inequality on both the macro and micro scale, the enabling conditions discussed below will only serve a specific group of rural actors; those who have the means to act upon the resulting opportunities.

A central policy implication is the need to properly connect the rural and urban regions through improving road and communication infrastructure and enabling social networks. Dispersed urbanisation patterns rather than centralised urban growth contribute to the potential number of rural people with access to cities. However, these overarching implications can only be realised when stable and inclusive institutions are present, providing local services and allowing for trade dynamics that contribute to rural livelihoods, rather than harm them.

## Footnotes

1 Exclusive Bangladesh
